# Bexarotene Reduces Production of CCL22 From Tumor-Associated Macrophages in Cutaneous T-Cell Lymphoma

**DOI:** 10.3389/fonc.2019.00907

**Published:** 2019-09-20

**Authors:** Kayo Tanita, Taku Fujimura, Yota Sato, Chunbing Lyu, Yumi Kambayashi, Dai Ogata, Satoshi Fukushima, Azusa Miyashita, Hideki Nakajima, Motoki Nakamura, Akimichi Morita, Setsuya Aiba

**Affiliations:** ^1^Department of Dermatology, Tohoku University Graduate School of Medicine, Sendai, Japan; ^2^Department of Dermatology, Saitama Medical University, Saitama, Japan; ^3^Department of Dermatology and Plastic Surgery, Faculty of Life Sciences, Kumamoto University, Kumamoto, Japan; ^4^Department of Dermatology, Kochi Medical School, Kochi University, Kochi, Japan; ^5^Department of Geriatric and Environmental Dermatology, Nagoya City University Graduate School of Medical Sciences, Nagoya, Japan

**Keywords:** advanced CTCL, bexarotene, tumor-associated macrophages, CCL22, immunomodulation

## Abstract

Bexarotene is a third-generation retinoid X receptor-selective retinoid that has been approved for use in the treatment of both early and advanced cutaneous T-cell lymphoma (CTCL). Although bexarotene has been used for decades in the treatment of CTCL, little is known about the mechanisms underlying its anti-tumor effects in CTCL patients. This study therefore focused on the immunomodulatory effects of bexarotene *in vivo* using an EL4 mouse T-cell lymphoma model, followed by investigation in CTCL patients treated with bexarotene. Intraperitoneal injection of bexarotene significantly decreased expressions of CCL22, CXCL5, CXCL10, and p19 in the tumor microenvironment. Based on those results, we then evaluated serum levels of CCL22, CXCL5, and CXCL10 in 25 patients with CTCL, revealing that CCL22 was significantly increased in advanced CTCL compared with early CTCL. Next, we evaluated serum levels of CCL22, CXCL5, and CXCL10 in CTCL patients treated with bexarotene. Serum levels of CCL22 were significantly decreased in 80% of CTCL patients who responded to bexarotene therapy. In addition, immunofluorescence staining revealed CD163^+^ M2 macrophages as the main source of CCL22. Moreover, bexarotene decreased the production of CCL22 by M2 macrophages generated from monocytes *in vitro*. Our findings suggest that the clinical benefits of bexarotene are partially attributable to suppressive effects on the production of CCL22 by M2-polarized tumor-associated macrophages.

## Introduction

Most cutaneous T-cell lymphomas (CTCLs) start as an indolent disease that progresses slowly, but finally advances to skin tumors followed by lymph node and visceral involvements (1). Since CTCL is a rare disease, and since established criteria for staging and response evaluation for CTCL are limited, few prospective clinical trials for advanced CTCL have been reported, and guidelines for the treatment of CTCL have yet to be established (2, 3). Instead, several preclinical studies have been used to determine the optimal therapy for CTCL (4–6). Among them, Shono et al. reported that mycosis fungoides (MF), the most common subtype of CTCL, shows high expression of CCR4 on the cell surface, correlating with poor prognosis of MF (4).

Moreover, Kim et al. reported that mogamulizumab therapy significantly prolonged progression-free survival (PFS) compared with vorinostat therapy for recurrent, advanced CTCL patients [hazard ratio, 0.53; 95% confidence interval (CI), 0.41–0.69; *p* < 0.0001) (3), and suggested immunotherapy as a promising option for the treatment of advanced CTCL. However, the optimal first-line therapy for advanced CTCL has remained unclear. Such reports suggested the importance of evaluating the production of CCR4 ligands CCL17 and CCL22 in the tumor microenvironment of CTCL. In addition, according to these preclinical studies, malignant T cells in CTCL have been shown to exhibit features of the regulatory T-cell (Treg) phenotype, Th2 phenotype, and Th17 phenotype (5), suggesting that not only Tregs and Th2-related factors, but also Th17-related factors are important in understanding the immunological background of CTCL.

Bexarotene is a third-generation retinoid X receptor (RXR)-selective retinoid that has been approved for use in the treatment of both early and advanced CTCL (7–9). Although bexarotene has been used for decades in the treatment of CTCL, and several preclinical studies have suggested anti-CTCL mechanisms are involved in the efficacy of this drug (10–12), little is known about the exact mechanisms underlying its anti-tumor effects in CTCL patients *in vivo* (7–9, 11, 12). Since bexarotene is useful for both early and advanced CTCL, bexarotene is applied in the real world to ultraviolet-tolerant early CTCL patients as a first-line therapy. Most such patients will subsequently need another type of therapy (2). Evaluating the immunological background of CTCL is therefore important, and this study focused on the immunomodulatory effects of bexarotene *in vivo* using an EL4 mouse T-cell lymphoma model, followed by investigation in CTCL patients treated with bexarotene.

## Materials and Methods

### Ethics Statement for Animal and Human Experiments

The protocol for the animal study was approved by the ethics committee at Tohoku University Graduate School of Medicine for Animal Experimentation, Sendai, Japan (permit number: 2017MdLMO-216). The research complied with the Tohoku University Graduate School of Medicine's Animal Experimentation Ethics guidelines and policies. All surgeries were performed under sodium pentobarbital anesthesia, and all efforts were made to minimize suffering. The protocol for the human study was approved by the ethics committee at Tohoku University Graduate School of Medicine, Sendai, Japan (permit number: 2018-1-772). All patients provided written informed consent to participate.

### Animals and T-Cell Lymphoma Cell Line

C57BL/6 mice (5–8 weeks old) were purchased from Japan Shizuoka Laboratory Animal Center (Shizuoka, Japan) and housed in the animal facility at Tohoku University Graduate School of Medicine. The EL4 murine T-cell lymphoma cell line was obtained from American Type Culture Collection (Manassas, VA) and cultured in Dulbecco's minimal essential medium supplemented with 10% heat-inactivated fetal calf serum. All mice were bred under specific pathogen-free conditions at Tohoku University Graduate School of Medicine.

### Tumor Inoculation and Treatment

EL4 T-cell lymphoma cells (100 μl of 2 × 10^6^ cells/ml) were subcutaneously injected into female C57BL/6 mice (13). For quantitative (q)RT-PCR and enzyme-linked immunosorbent assay (ELISA), 0.15 mg bexarotene was intraperitoneally injected on day 12, and the tumor was harvested on day 14. For qRT-PCR, the whole tumor was frozen with liquid nitrogen, then crushed with a Cryo-Press (MICROTEC, Chiba, Japan), as described previously (14). Total RNA was extracted using ISOGEN (NIPPON GENE, Tokyo, Japan) according to the manufacturer's instructions. For the therapeutic experiments, we measured the size of established tumors with calipers (Mitsutoyo, Utsunomiya, Japan) and estimated tumor volume using the following formula: π/6 × length × width (14). Starting on day 6, we intraperitoneally injected 0.15 mg of bexarotene or 0.30 mg of anti-CCL22 antibodies (R&D Systems, Minneapolis, MN) on day 6 and day 12. Tumor-bearing animals were sacrificed when the tumor resulted in severe ulceration or reached a size of 1,000 mm^3^.

### RNA Extraction and Quantitative Real-Time PCR Experiments

Total RNA was extracted using an RNeasy Micro kit (Qiagen, Courtaboeuf, France) in accordance with the manufacturer's instructions. RNA was eluted with 14 μl of RNase-free water. DNase I treatment (RNase-Free DNase Set; Qiagen) was performed to remove contaminating genomic DNA. Reverse transcription was performed with the SuperScript VILO cDNA Synthesis kit (Invitrogen, Carlsbad, CA). Amplification reactions were performed using an Mx 3000P Real-Time Quantitative PCR System (Stratagene, San Diego, CA). Relative mRNA expression levels were calculated for each gene and each time point after normalization against GAPDH using the ΔCt method or ΔΔCt method.

### Patients

Data from 25 CTCL patients were collected from five clinical sites in Japan. Pathologists and dermatologists in each institute had diagnosed these patients with CTCL both clinically and pathologically. No patients who were administered bexarotene had received any systemic therapies previously. We have summarized the clinical information in Table 1.

**Table 1:** Characteristics of patients with CTCL.

	**Age**	**Sex**	**Subtype**	**Stage**	**Therapy**	**Response for bexarotene**	**CCR4**
Case 1	46	F	MF	T3N0M0B0 stage IIB	Bexarotene	PR	+
Case 2	46	M	MF	T3N0M0B0 stage IIB	Bexarotene	PR	+
Case 3	37	M	MF	T1aN0M0B0 stage IA	NB-UVB	N.A.	N.A.
Case 4	45	M	PCPTCL	T3bN1M0 stage IIIA	Bexarotene	SD	–
Case 5	44	M	MF	T4N0M0B0 Stage IIIA	Bexarotene	PD	+
Case 6	60	M	MF	T2bN0M0B0 stage IB	Bexarotene	PD	+
Case 7	81	M	ALCL	T3bN0M0 ALCL stage IIIB	Bexarotene	PD	N.A.
Case 8	78	M	MF	T2bN0M0 stage IB	Bexarotene	CR	N.A.
Case 9	84	F	MF	T1bN0M0 stage IA	Bexarotene	PR	N.A.
Case 10	51	F	MF	T3N3M0B0 stage IVA2	Bexarotene	PD	+
Case 11	75	F	MF	T1aN0M0 stage IA	Topical steroid	N.A.	N.A.
Case 12	67	M	MF	T2bN0M0 stage IB	NB-UVB	N.A.	N.A.
Case 13	44	M	MF	T1bN3M0 Stage IVA2	Bexarotene	PR	+
Case 14	48	F	LyP	T2aN0M0 stage IIA	Bexarotene	CR	–
Case 15	63	F	MF	T2bNxM0B0 stage IIA	NB-UVB	N.A.	+
Case 16	26	F	PCALCL	T1bN0M0 stage IA	Topical steroid	N.A.	–
Case 17	86	F	ATLL	stage IV (Ann Arbor)	Bexarotene	PR	+
Case 18	69	M	MF	T3N0M0 stage IIB	Bexarotene	PR	+
Case 19	67	F	PCPTCL	T3aN3M0B0 stage IIIB	Bexarotene	CR	–
Case 20	76	M	NKTL	T3bNxM0B0 stage IIIA	Bexarotene	PD	–
Case 21	70	M	MF	T3N0M0B0 stage IIB	Bexarotene	PD	+
Case 22	42	F	MF	T4N0M0 Stage IIIA	Bexarotene	PD	+
Case 23	70	M	MF	T2bN0M0B0 Stage IB	Bexarotene	PR	+
Case 24	59	M	MF	T1aN0M0B0 stage IA	Topical steroid	N.A.	N.A.
Case 25	62	F	MF	T3N0M0 stage IIB	Bexarotene plus NB-UVB	PR	+

*MF, mycosis fungoides; PCPTCL, primary cutaneous peripheral T-cell lymphoma; ALCL, anaplastic large-cell lymphoma; PCALCL, primary cutaneous anaplastic large-cell lymphoma; ATLL, adult T-cell leukemia/lymphoma; NKTL, natural killer T-cell lymphoma; NB-UVB, narrowband ultraviolet B*.

### Reagents

The following antibodies were used for immunofluorescence (IF): mouse anti-human CD163 phycoerythrin-conjugated monoclonal antibody (R&D Systems), rabbit polyclonal anti-CCL22 antibody (Biorbyt, Cambridge, UK), rabbit polyclonal anti-CXCL5 antibody (Lifespan Bioscience, Seattle, WA), mouse anti-CXCL10 antibody (Lifespan Bioscience), Alexa Fluor 488-conjugated anti-mouse rat immunoglobulin (Ig)G (Abcam, Tokyo, Japan), and Alexa Fluor 488-conjugated anti-rabbit goat IgG (Abcam). We used the following antibodies for immunohistochemical staining: rabbit polyclonal antibodies for human CCL22 (R&D Systems) and human CXCL5 (LifeSpan Bioscience).

### Tissue Samples and Immunohistochemical Staining

Each sample was processed for single staining of CCL22 and CXCL5, and developed with liquid permanent red (DAKO, Santa Clara, CA). Briefly, formalin-fixed, paraffin-embedded tissue samples were sectioned at 4 μm and deparaffinized. After protease treatment for antigen retrieval, sections were blocked with goat serum for 10 min, then exposed to primary antibodies at 4°C overnight. Sections were developed with 3-Amino-9-ethylcarbazole (AEC).

### Tissue Samples and IF Staining

For cryosections, each sample was frozen in optimal cutting temperature embedding medium, and 6-μm sections were fixed with cold acetone for 10 min and blocked with IF buffer (PBS, 5% bovine serum albumin). Each section was therefore incubated with relevant antibodies. Slides were mounted in DAPI Fluoromount-G (Southern Biotech, Birmingham, AL) and examined using an LSM 700 microscope equipped with a SPOT digital camera (Zeiss, Oberkochen, Germany).

### Cytokine ELISA

Secretion of CCL22 in each tumor was determined using ELISA kits (R&D Systems), according to the manufacturer's instructions. Serum levels of CCL22, CXCL5, and CXCL10 in each CTCL patient were measured according to the manufacturer's instructions. For patients treated with bexarotene, serum was obtained at 0 days and/or 4 weeks after bexarotene administration (300 mg/m^2^), then stored for ELISA analysis of serum levels of each chemokine. For peripheral blood monocytes (PBMo)-derived M2 macrophages, after the 7-day culture, supernatants were collected and secretion of CCL22, CXCL5, and sCSD163 was determined by ELISA kits (R&D Systems), according to the manufacturer's instructions.

Data from each donor were obtained as the mean of duplicate assays.

### Culture of M2 Macrophages From Human Peripheral Blood Monocytes

CD14^+^ monocytes were isolated from peripheral blood mononuclear cells from healthy donors using MACS beads (CD14 microbeads; Miltenyi Biotec, Sunnyvale, CA) according to the manufacturer's protocol. CD14^+^ monocytes (2 × 10^5^/ml) were cultured in complete medium containing 100 ng/ml of recombinant human M-CSF for 5 days, as previously reported (15, 16). On day 5, monocyte-derived macrophages were treated with recombinant human IL-4 (20 ng/ml) with or without bexarotene (10 ng/ml−1 μg/ml) for 48 h, and culture supernatant was harvested.

### Flow Cytometry

The surface expression of CD163 and arginase 1 on macrophages was analyzed by flow cytometry. Cell staining was conducted with PE-conjugated anti-CD163 (R & D system), FITC-conjugated anti-arginase 1 (R & D system), PE-conjugated isotype control Ab (BD Bioscience, Tokyo, Japan), or FITC-conjugated isotype control Ab (BD Bioscience). The cells were analyzed with a C6 flow cytometer (Acuri Cytometers Inc., Ann Arbor, MI).

### Statistical Analysis

For a single comparison of two groups, the Mann–Whitney *U*-test was used. The level of significance was set at *p* < 0.05.

## Results

### Immunomodulatory Effects of Intraperitoneal Injection of Bexarotene on the Tumor Microenvironment of EL4 T-Cell Lymphoma

**Figure 1:**
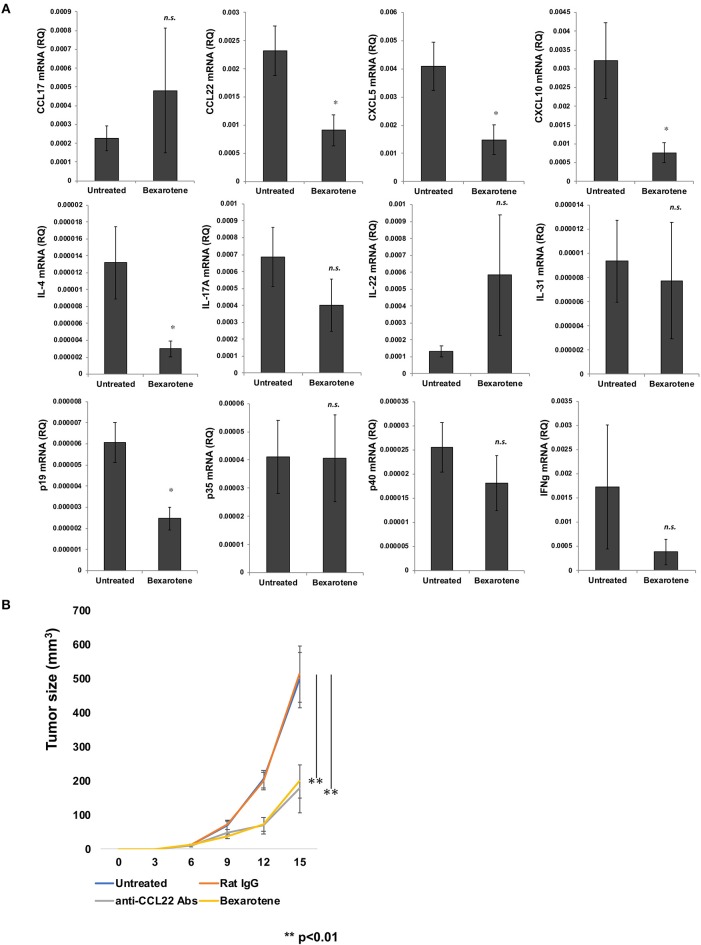
Intraperitoneally administered bexarotene modifies chemokine and cytokine expression in mouse EL4 T-cell lymphoma. Expression of chemokines and cytokines in EL4 T-cell lymphoma was analyzed by quantitative RT-PCR using the ΔCt method (*n* = 5). Averages of five independent experiments are shown **(A)**. We subcutaneously injected 100 μl of 2 × 10^6^ cells/ml of EL4 T-cell lymphoma cells, and intraperitoneally injected 0.15 mg bexarotene or 0.30 mg anti-CCL22 antibodies (R&D Systems, Minneapolis, MN) at day 6 and day 12 (*n* = 5 for each treated group) Error bar represents ± standard deviation. **(B)** One representative experiment of two is shown *p < 0.05, **p < 0.01. Error bar represents ± standard deviation.

Since the immunological microenvironment of CTCL resembles that of atopic dermatitis (16–18), and as tumor-associated macrophages (TAMs) have been reported to play a significant role in stimulating the developing tumor microenvironment by periostin and IL-4 in lesional skin of mycosis fungoides (MF) (16), we hypothesized that bexarotene might affect the immunological functions of TAMs in tumor sites of CTCL. To investigate the immunomodulatory effects of bexarotene on the tumor microenvironment *in vivo*, we used the mouse EL4 T-cell lymphoma model. First, we evaluated TAM-related chemokines, Th1/Th2-related cytokines and proinflammatory cytokines. Intraperitoneal administration of bexarotene significantly decreased expressions of CCL22, CXCL5, CXCL10, IL-4, and p19 mRNA in the tumor microenvironment (Figure 1A). No significant differences were seen in the expressions of CCL17, IL-17A, p35, or p40 mRNA.

### Bexarotene Suppresses the Growth of EL4 T-Cell Lymphoma *in vivo*

Since bexarotene significantly decreased expression of CCL22 mRNA in the tumor microenvironment, we hypothesized that bexarotene, as well as anti-CCL22 antibody (Ab) could suppress the growth of EL-4 T cell lymphoma *in vivo*. We examined the therapeutic effects of bexarotene *in vivo* using the EL-4 murine T-cell lymphoma model. We treated EL-4 murine T-cell lymphoma (3–4 mm in diameter) on the backs of mice by intraperitoneal injection of bexarotene (0.15 mg/mouse) or anti-CCL22 Ab (0.30 mg/mouse) on days 6 and 12. For the control antibody, we used rat IgG (0.30 mg/mouse). Both bexarotene and anti-CCL22 Ab significantly suppressed the growth of EL-4 T-cell lymphoma (Figure 1B).

### Serum Levels of CCL22, CXCL5, and CXCL10 in Patients With CTCL

**Figure 2:**
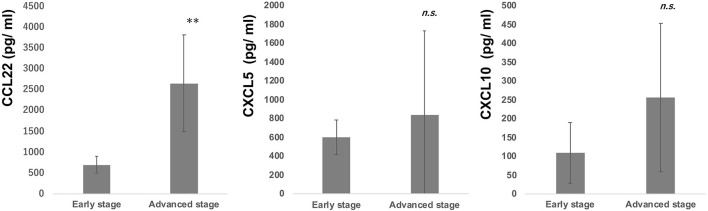
Serum levels of CCL22, CXCL5, and CXCL10 in early and advanced CTCL patients. Serum levels of CCL22, CXCL5, and CXCL10 were examined by ELISA in 9 early CTCL patients and 16 advanced CTCL patients. **p < 0.01, Student's *t*-test; *n.s*., not significant. Error bar represents ± standard deviation.

**Figure 3:**
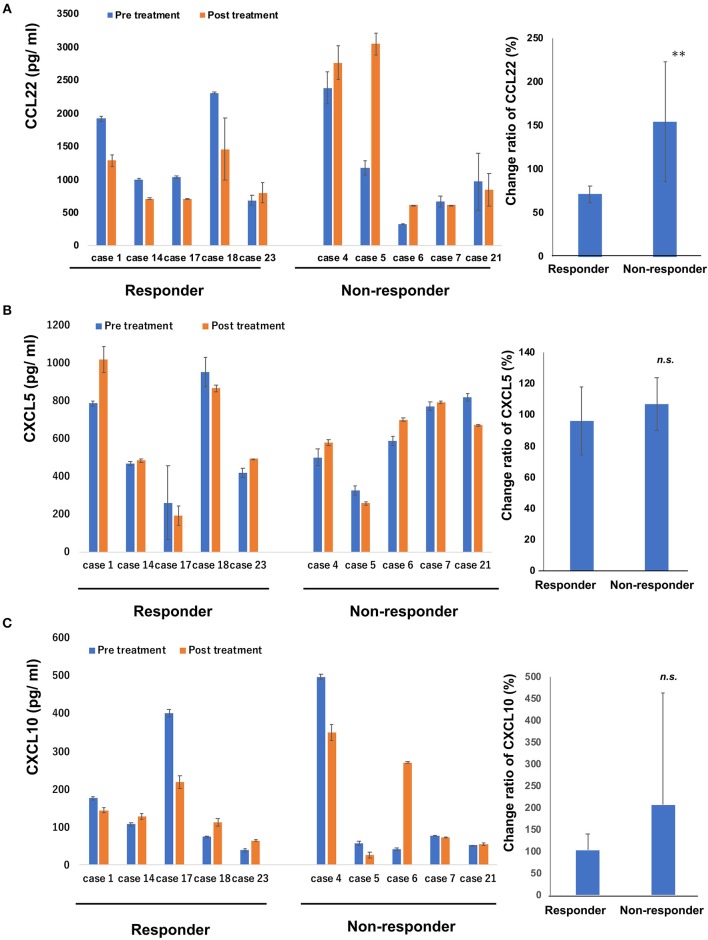
Serum levels of CCL22, CXCL5 and CXCL10 in patients treated with bexarotene. Serum levels of CCL22 **(A)**, CXCL5 **(B)**, and CXCL10 **(C)** in responders (*n* = 5) and non-responders (*n* = 5) at day 0 and day 28 were measured by ELISA. Error bar represents ± standard deviation. Change ratio is calculated described below: (post-treatment serum chemokine)/(pre-treatment serum chemokine level) × 100 (%). Change ratios of serum CCL22 **(A)**, CXCL5 **(B)**, and CXCL10 **(C)** in CTCL are calculated in each sample and the average are shown. Error bar represents ± standard deviation. **p < 0.01, Mann–Whitney *U*-test; *n.s*., not significant.

Since intraperitoneal injection of bexarotene decreased expression of CCL22, CXCL5, and CXCL10 mRNA in EL4 mouse T-cell lymphoma, we hypothesized that serum levels of CCL22, CXCL5, or CXCL10 might be associated with response in CTCL patients treated with bexarotene. We therefore first evaluated serum levels of CCL22, CXCL5, and CXCL10 in 9 patients with early CTCL and 16 patients with advanced CTCL (Table 1). Serum levels of CCL22 were significantly increased in advanced CTCL compared with early CTCL (Figure 2). In contrast, no significant difference in serum levels of CXCL5 and CXCL10 were seen between early and advanced CTCL (Figure 2). Next, we evaluated serum levels of CCL22, CXCL5, and CXCL10 before and after administration of oral bexarotene. All patients were administered bexarotene at 300 mg/m^2^/day. Serum levels of CCL22 were decreased in CTCL patients who responded to bexarotene therapy, but were increased in CTCL patients who showed progressive disease 4 weeks after starting bexarotene therapy (Figure 3A). The change ratio of serum CCL22 in CTCL patients who responded to bexarotene therapy was significantly lower than that in CTCL patients who showed progressive disease by 4 weeks after starting bexarotene therapy (Figure 3A). In contrast to serum CCL22, no differences were identified in the change ratios of serum CXCL5 (Figure 3B) or CXCL10 (Figure 3C) among responders and non-responders to bexarotene therapy.

### CD163^+^ TAMs Produce CCL22, but Not CXCL5 and CXCL10 in Patients With MF

**Figure 4:**
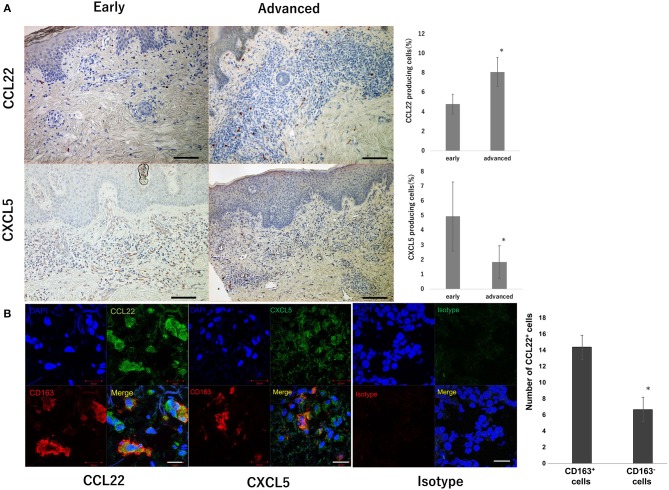
CCL22 producing cells in the lesional skin of MF. Representative paraffin-embedded tissue samples from the lesional skin at each stage of MF. Sections were deparaffinized and stained using anti-CCL22 or anti-CXCL5 antibodies. Three representative high-power fields of each section were selected from dermis associated with a dense dermal lymphoid infiltrate. Sections were developed with 3-Amino-9-ethylcarbazole. The percentage of CCL22^+^ and CXCL5^+^ cells was calculated as follows: CCL22^+^ cells or CXCL5^+^ cells/hematoxylin-positive cells × 100. Quantification of percentages of CCL22^+^ cells and CXCL5^+^ cells from 9 early CTCL patients and 16 advanced CTCL patients is shown. Scale bar, 100 μm. Error bar represents ± standard deviation **(A)**. IF staining of CTCL for CCL22 (green), CD163 (red), and DAPI (blue, nuclei), and CXCL5 (green), CD163 (red), and DAPI (blue, nuclei). A merged image is also shown, with green and red combining into yellow. The isotype control IgG1 stains as red or green. Scale bar, 20 μm **(B)**. Representative specimens from three cases are shown. The number of immunoreactive cells was counted at a magnification of × 400. The average of three independent fields in each sample was calculated (*p < 0.05). Error bar represents ± standard deviation.

Since oral intake of bexarotene decreased serum CCL22 in CTCL patients, we next investigated source cells of CCL22, CXCL5, and CXCL10 in the lesional skin of MF, as the largest subtype of CTCL. The number of CCL22-producing cells significantly increased in advanced stages compared to that in the early stage (Figure 4A). In contrast, the number of CXCL5-producing cells significantly decreased in parallel with MF stage (Figure 4A). CXCL10-producing cells were not detected in the lesional skin of MF (data not shown). IF staining revealed that CD163^+^ M2 macrophages mainly produced CCL22 (Figure 4B), but a few CD163^+^ M2 macrophages produced CXCL5 in the lesional skin of MF (Figure 4B).

### Bexarotene Decreased Production of CCL22 From Monocyte-Derived M2 Macrophages *in vitro*

**Figure 5:**
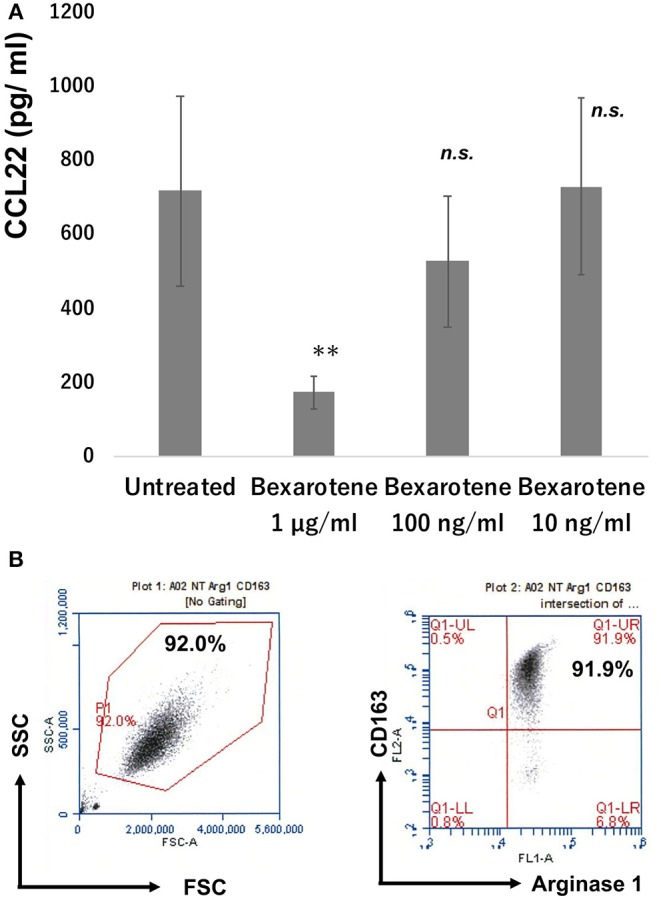
Production of CCL22 from M2 macrophages treated with bexarotene. Culture supernatant from M2 macrophages was harvested as described in section Materials and Methods and measured by ELISA (*n* = 3). Data from each donor were obtained from triplicate assays, and mean ± SD was calculated. Representative data from at least three independent experiments are shown. **p < 0.01, Mann–Whitney *U*-test; *n.s*., not significant. Error bar represents ± standard deviation **(A)**. M-CSF-induced M2 macrophages from PBMCs: the expression of CD163 and arginase 1 was examined by flow cytometry **(B)**.

Since CD163^+^ TAMs produced CCL22 in the lesional skin of MF, we hypothesized that bexarotene might decrease the production of CCL22 from CD163^+^ M2 macrophages. To test this, we evaluated the production of chemokines from CD163^+^ M2 macrophages using M2 macrophages generated from peripheral blood mononuclear cells (PBMCs) in healthy donors (15). Production of CCL22 was significantly decreased by bexarotene in a dose-dependent manner (Figure 5A). The purity of cultured CD163+ M2 macrophages is >90% as assessed by FACS analysis (Figure 5B).

## Discussion

Since most CTCL treatment requires subsequent additional therapy (2), evaluation of the immunological background of CTCL is important. For example, Richardson et al. reported that bexarotene reduced the expression of chemokine receptors to suppress the chemotaxis of CTCL cells to CCL17 *in vitro* (12). They concluded that bexarotene might inhibit migration of CTCL cells to the skin by suppressing CCR4 expression (12). In other reports, bexarotene selectively induced CTCL lineages to increase integrin β7 expression and function prior to growth arrest and apoptosis *in vitro* (11). Although those reports partially explain the efficacy of bexarotene for CTCL patients, direct evidence for the immunomodulatory effects of bexarotene is lacking. Since most CTCL treatment requires subsequent additional therapy (2), evaluation of the immunological background of CTCL is important.

TAMs create an immunosuppressive tumor microenvironment by producing various chemokines that attract other immunosuppressive cells such as Tregs, myeloid-derived suppressor cells (MDSCs), or even attract CTCL cells to establish the tumor microenvironment (12, 16, 19). In CTCL, at least, periostin and IL-4 could determine the functional maturation of TAMs, leading to development of the characteristic microenvironment of CTCL in each stage (16). Notably, administration of IFN-α or IFN-γ, both of which have been approved for use in the treatment of CTCL, has been shown to modulate the chemokine profiles of TAMs in the lesional skin of CTCL (20), suggesting that re-polarization of TAMs into anti-tumor macrophages might be one of the possible mechanisms of anti-CTCL drugs. In the present study, IF staining suggested the possible source of CCL22 is CD163^+^ TAMs, and bexarotene decreased CCL22 production from CD163^+^ M2 macrophages *in vitro*, suggesting that bexarotene induces anti-CTCL effects by suppressing CCL22 production from TAMs in CTCL patients.

CCL22 diverts Tregs and controls B16F10 melanoma growth (21, 22). Indeed, intratumoral administration of anti-CCL22 antibody inhibited B16F10 melanoma growth by decreasing Treg recruitment at the tumor site (21), suggesting that a reduction in tumor-derived CCL22 could suppress melanoma growth. In CTCL, Chang et al. reported that anti-CCR4 antibody significantly suppressed MAC-1 mouse CTCL growth *in vivo* by inhibiting CCR4/CCL22 pathways and antibody-dependent cellular cytotoxicity activities (22). Since CCL22 attracts CCR4^+^ lymphocytes, such as CTCL cells, Tregs, and Th2 cells (23), the decrease in CCL22 might suppress the development of tumor mass *in vivo*. Indeed, in this study, bexarotene decreased serum CCL22 in 80% of CTCL patients who responded clinically to bexarotene, but did not decrease serum CCL22 in any of the CTCL patients who were not clinically responsive to bexarotene. These data suggested that CCL22 could provide a biomarker to evaluate the efficacy of bexarotene in patients with CTCL.

Since intraperitoneal injection of bexarotene decreased CXCL5 and CXCL10 mRNA expression in EL4 mouse T-cell lymphoma *in vivo*, we evaluated serum levels of these chemokines in patients with early and advanced CTCL. Unlike CCL22, no differences in serum levels of CXCL5 and CXCL10 were identified between early and advanced CTCL. Moreover, no differences in serum levels of CXCL5 and CXCL10 were identified between responder and non-responder patients. Although both CXCL5 and CXCL10 are important for immunosuppression in the tumor microenvironment by recruiting polymorphonuclear MDSCs, neutrophils, Tregs, and effector T cells in solid tumor such as melanoma, renal cell carcinoma, esophageal carcinoma, and pancreatic cancer (23–27), bexarotene did not affect serum levels of CXCL5 and CXCL10 in the present study. Since no significant difference was identified in serum levels of CXCL5 and CXCL10 between early and advanced CTCL, these chemokines might not directly affect CTCL progression.

This study investigated the immunomodulatory effects of bexarotene *in vivo* using an EL4 mouse T-cell lymphoma model, followed by CTCL patients treated with bexarotene. Our findings suggested the clinical benefit of bexarotene is partially explained by the suppressive effects on the production of CCL22 from M2-polarized TAMs, which should contribute to the recruitment of CTCL cells, Tregs, and Th2 cells in the lesional skin of CTCL (Supplementary Figure 1).

## Data Availability

The raw data supporting the conclusions of this manuscript will be made available by the authors, without undue reservation, to any qualified researcher.

## Ethics Statement

The protocol for the animal study was approved by the ethics committee at Tohoku University Graduate School of Medicine for Animal Experimentation, Sendai, Japan (permit number: 2017MdLMO-216). The research complied with the Tohoku University Graduate School of Medicine's Animal Experimentation Ethics guidelines and policies. All surgeries were performed under sodium pentobarbital anesthesia, and all efforts were made to minimize suffering. The protocol for the human study was approved by the ethics committee at Tohoku University Graduate School of Medicine, Sendai, Japan (permit number: 2018-1-772). All patients provided written informed consent to participate.

## Author Contributions

TF designed the research study. KT, TF, YS, and CL performed experiments with the mouse model. KT, TF, YS, CL, and YK gathered and analyzed the human data. TF, YK, DO, SF, AMi, HN, MN, and AMo treated the patients and acquired the clinical data and samples. TF wrote the manuscript. TF and SA supervised the study.

### Conflict of Interest Statement

The authors declare that the research was conducted in the absence of any commercial or financial relationships that could be construed as a potential conflict of interest.

## Supplementary Material

Supplementary Figure 1 Bexarotene suppresses the recruitment of CTCL as well as Tregs and Th2 by the reduction of CCL22 from TAMs.

## References

[1] Willemze R Jaffe ES Burg G Cerroni L Berti E Swerdlow SH. WHO-EORTC classification for cutaneous lymphomas. Blood. (2005) 105:3768–85. 10.1182/blood-2004-09-3502 15692063

[2] Quaglino P Maule M Prince HM Porcu P Horwitz S Duvic M. Global patterns of care in advanced stage mycosis fungoides/Sezary syndrome: a multicenter retrospective follow-up study from the Cutaneous Lymphoma International Consortium. Ann Oncol. (2017) 28:2517–25. 10.1093/annonc/mdx352 28961843

[3] Kim YH Bagot M Pinter-Brown L Rook AH Porcu P Horwitz SM. Mogamulizumab versus vorinostat in previously treated cutaneous T-cell lymphoma (MAVORIC): an international, open-label, randomised, controlled phase 3 trial. Lancet Oncol. (2018) 19:1192–204. 10.1016/S1470-2045(18)30379-6 30100375

[4] Shono Y Suga H Kamijo H Fujii H Oka T Miyagaki T. Expression of CCR3 and CCR4 suggests a poor prognosis in mycosis fungoides and Sézary syndrome. Acta Derm Venereol. (2019) 99:809–12. 10.2340/00015555-3207 31045236

[5] Rubio Gonzalez B Zain J Rosen ST Querfeld C. Tumor microenvironment in mycosis fungoides and Sézary syndrome. Curr Opin Oncol. (2016) 28:88–96. 10.1097/CCO.0000000000000243 26632770

[6] Ehrentraut S Schneider B Nagel S Pommerenke C Quentmeier H Geffers R. Th17 cytokine differentiation and loss of plasticity after SOCS1 inactivation in a cutaneous T-cell lymphoma. Oncotarget. (2016) 7:34201–16. 10.18632/oncotarget.9077 27144517 PMC5085149

[7] Zhang C Hazarika P Ni X Weidner DA Duvic M. Induction of apoptosis by bexarotene in cutaneous T-cell lymphoma cells: relevance to mechanism of therapeutic action. Clin Cancer Res. (2002) 8:1234–40. 12006543

[8] Duvic M Hymes K Heald P Breneman D Martin AG Myskowski P. Bexarotene is effective and safe for treatment of refractory advanced-stage cutaneous T-cell lymphoma: multinational phase II-III trial results. J Clin Oncol. (2001) 19:2456–71. 10.1200/JCO.2001.19.9.2456 11331325

[9] Scarisbrick JJ Morris S Azurdia R Illidge T Parry E Graham-Brown R. U.K. consensus statement on safe clinical prescribing of bexarotene for patients with cutaneous T-cell lymphoma. Br J Dermatol. (2013) 168:192–200. 10.1111/bjd.12042 22963233

[10] Chou CF Hsieh YH Grubbs CJ Atigadda VR Mobley JA Dummer R. The retinoid X receptor agonist, 9-cis UAB30, inhibits cutaneous T-cell lymphoma proliferation through the SKP2-p27kip1 axis. J Dermatol Sci. (2018) 90:343–56. 10.1016/j.jdermsci.2018.03.006 29599065 PMC6329374

[11] Wang L DeMarco SS Chen J Phillips CM Bridges LC. retinoids bias integrin expression and function in cutaneous T-cell lymphoma. J Invest Dermatol. (2015) 135:2102–8. 10.1038/jid.2015.122 25826424

[12] Richardson SK Newton SB Bach TL Budgin JB Benoit BM Lin JH. Bexarotene blunts malignant T-cell chemotaxis in Sezary syndrome: reduction of chemokine receptor 4-positive lymphocytes and decreased chemotaxis to thymus and activation-regulated chemokine. Am J Hematol. (2007) 82:792–7. 10.1002/ajh.20952 17546636

[13] Fujimura T Ring S Umansky V Mahnke K Enk AH Regulatory T cells (Treg) stimulate B7-H1 expression in myeloid derived suppressor cells (MDSC) in *ret* melanomas. J Invest Dermatol. (2012) 132:1239–46. 10.1038/jid.2011.416 22189788

[14] Kakizaki A Fujimura T Furudate S Kambayashi Y Yamauchi T Yagita H Immunomodulatory effect of peritumoral administration of interferon-beta on melanoma through tumor-associated macrophages. Oncoimmunology. (2015) 4:e1047584 10.1080/2162402X.2015.1047584 26451326 PMC4589056

[15] Fujimura T Kambayashi Y Furudate S Asano M Kakizaki A Aiba S Receptor activator of nuclear factor k-B ligand (RANKL) promotes the production of CCL17 from RANK+ M2 macrophages. J Invest Dermatol. (2015) 135:2884–7. 10.1038/jid.2015.209 26053051

[16] Furudate S Fujimura T Kakizaki A Kambayashi Y Asano M Watabe A. The possible interaction between periostin expressed by cancer stroma and tumor-associated macrophages in developing mycosis fungoides. Exp Dermatol. (2016) 25:107–12. 10.1111/exd.12873 26441016

[17] Masuoka M Shiraishi H Ohta S Suzuki S Arima K Aoki S. Periostin promotes chronic allergic inflammation in response to Th2 cytokines. J Clin Invest. (2012) 122:2590–600. 10.1172/JCI58978 22684102 PMC3386810

[18] Ando T Xiao W Gao P Namiranian S Matsumoto K Tomimori Y. Critical role for mast cell Stat5 activity in skin inflammation. Cell Rep. (2014) 6:366–76. 10.1016/j.celrep.2013.12.029 24412367 PMC4329986

[19] Fujimura T Kambayashi Y Fujisawa Y Hidaka T Aiba S. Tumor-associated macrophages: therapeutic targets for skin cancer. Front Oncol. (2018) 8:3. 10.3389/fonc.2018.00003 29410946 PMC5787130

[20] Furudate S Fujimura T Kakizaki A Hidaka T Asano M Aiba S Tumor-associated M2 macrophages in mycosis fungoides acquired immunomodulatory function by interferon alpha and interferon gamma. J Dermatol Sci. (2016) 83:182–9. 10.1016/j.jdermsci.2016.05.004 27342040

[21] Furudate S Fujimura T Kambayashi Y Kakizaki A Hidaka T Aiba S. Immunomodulatory effect of imiquimod through CCL22 produced by tumor-associated macrophages in B16F10 melanomas. Anticancer Res. (2017) 37:3461–71. 10.21873/anticanres.11714 28668835

[22] Chang DK Sui J Geng S Muvaffak A Bai M Fuhlbrigge RC. Humanization of an anti-CCR4 antibody that kills cutaneous T-cell lymphoma cells and abrogates suppression by T-regulatory cells. Mol Cancer Ther. (2012) 11:2451–61. 10.1158/1535-7163.MCT-12-0278 22869555 PMC3496034

[23] Forsthuber A Lipp K Andersen L Ebersberger S Graña-Castro Ellmeier W. CXCL5 as regulator of neutrophil function in cutaneous melanoma. J Invest Dermatol. (2019) 139:186–94. 10.1016/j.jid.2018.07.006 30009831

[24] Soler-Cardona A Forsthuber A Lipp K Ebersberger S Heinz M Schossleitner K. CXCL5 facilitates melanoma cell-neutrophil interaction and lymph node metastasis. J Invest Dermatol. (2018) 138:1627–35. 10.1016/j.jid.2018.01.035 29474942

[25] Najjar YG Rayman P Jia X Pavicic PG Jr Rini BI Tannenbaum C. Myeloid-derived suppressor cell subset accumulation in renal cell carcinoma parenchyma is associated with intratumoral expression of IL1β, IL8, CXCL5, and Mip-1α. Clin Cancer Res. (2017) 23:2346–55. 10.1158/1078-0432.CCR-15-1823 27799249 PMC5411325

[26] Lunardi S Lim SY Muschel RJ Brunner TB. IP-10/CXCL10 attracts regulatory T cells: implication for pancreatic cancer. Oncoimmunology. (2015) 4:e1027473. 10.1080/2162402X.2015.1027473 26405599 PMC4570127

[27] Lu L Pan K Zheng HX Li JJ Qiu HJ Zhao JJ. IL-17A promotes immune cell recruitment in human esophageal cancers and the infiltrating dendritic cells represent a positive prognostic marker for patient survival. J Immunother. (2013) 36:451–8. 10.1097/CJI.0b013e3182a802cf 23994890

