# Supplementary figures and images for: Bexarotene Reduces Production of CCL22 From Tumor-Associated Macrophages in Cutaneous T-Cell Lymphoma
Source: Front Oncol. 2019 Sep 20;9:907. doi: 10.3389/fonc.2019.00907 (PMC6763730; doi:10.3389/fonc.2019.00907)

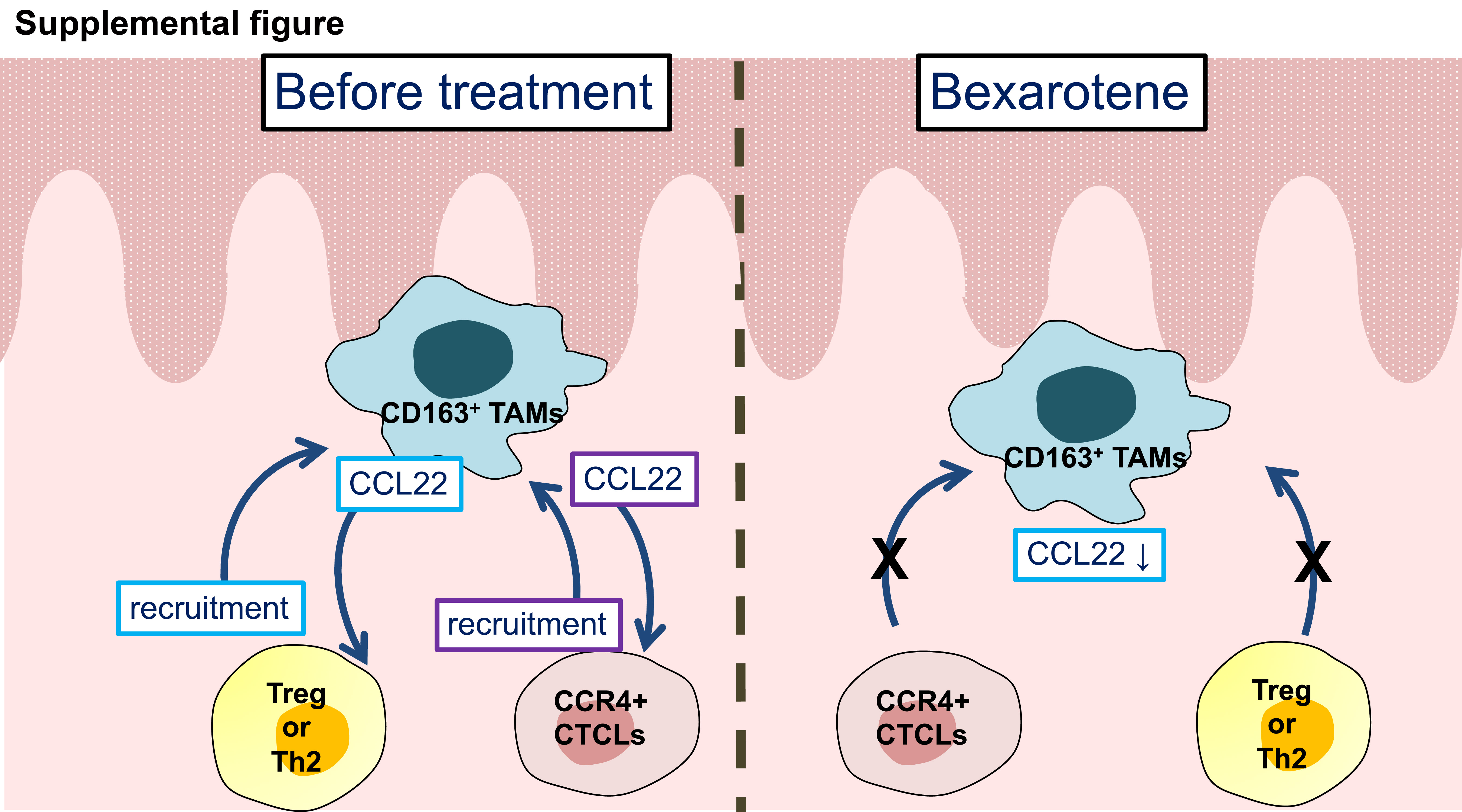

Supplement: Supplementary Figure 1 — Bexarotene suppresses the recruitment of CTCL as well as Tregs and Th2 by the reduction of CCL22 from TAMs. [file Image_1.JPEG]
